# Alcohol-Related Elevation of Liver Transaminase Is Associated With Gut Microbiota in Male

**DOI:** 10.3389/fmed.2022.823898

**Published:** 2022-02-22

**Authors:** Mengfan Jiao, Su Yan, Qingmiao Shi, Ying Liu, Yaoguang Li, Jun Lv, Suying Ding, Ang Li

**Affiliations:** ^1^Department of Infectious Diseases, The First Affiliated Hospital of Zhengzhou University, Zhengzhou, China; ^2^Gene Hospital of Henan Province, The First Affiliated Hospital of Zhengzhou University, Zhengzhou, China; ^3^Health Management Center, The First Affiliated Hospital of Zhengzhou University, Zhengzhou, China; ^4^College of Public Health, Zhengzhou University, Zhengzhou, China; ^5^State Key Laboratory for Diagnosis and Treatment of Infectious Diseases, First Affiliated Hospital, School of Medicine, Zhejiang University, Hangzhou, China; ^6^National Clinical Research Center for Infectious Diseases, First Affiliated Hospital, School of Medicine, Zhejiang University, Hangzhou, China

**Keywords:** gut microbiome, alcohol, liver transaminase, whole-genome sequencing, individual susceptibility, inflammation

## Abstract

Alcoholic liver damage has become a widespread health problem as alcohol consumption increases and is usually identified by elevated liver transaminase. We conducted this study to investigate the role of the gut microbiome in the individual susceptibility to alcoholic liver injury. We divided the participants into four groups based on alcohol consumption and liver transaminase elevation, which were drinking case group, drinking control group, non-drinking case group, and non-drinking control group. The drinking case group meant participants who were alcohol consumers with elevated liver transaminase. We found that alpha and beta diversities of the drinking case group differed from the other three groups. Species *Faecalibacterium prausnitzii* and *Roseburia hominis* were significantly in lower abundance in the drinking case group and were proved the protective effect against inflammatory liver damage in the former study. *Ruminococcus gnavus* exhibited the most positive association to alanine aminotransferase (ALT) and aspartate aminotransferase (AST) and contributed to liver inflammation.

## Introduction

Alcohol abuse is an important public risk factor for a variety of health problems and is related to more than 200 diseases, among which the liver is a major target organ (1–3). Alanine aminotransferase (ALT) and aspartate aminotransferase (AST) are the most commonly used and the most easily available liver injury serum markers (4). When hepatocyte necrosis occurs, ALT and AST can be released into the blood circulation, resulting in increased levels of serum ALT and AST (5).

The earlier investigations uncovered several mechanisms of alcohol-induced liver injury. Ethanol-induced oxidative stress (6) and the toxic product of ethanol metabolism acetaldehyde were found to contribute to liver damage (7). Notably, the emerging evidence confirmed the importance of gut microbial dysbiosis in the progress of alcoholic liver damage. The altered colonic microbiome was observed in alcoholism: the gut microbiome dysbiosis in alcoholic liver disease is long-lasting and serum endotoxin levels were higher than the healthy individuals (8). Alcohol can improve intestinal permeability through transepithelial permeability and paracellular permeability (9). The elevated intestinal permeability, namely a gut leaky, is associated with liver injury, which leads to the translocation of bacterial toxins into the bloodstream (10, 11). Endotoxin can enter the liver through the blood circulation, activate Kupffer cells, and induce liver damage (12).

Individual susceptibility plays an important role in hepatocyte necrosis (13). Only about 35% of those who have alcohol use disorders develop early liver disease. Many risk factors contribute to liver damage susceptibility. Felix Stickel et al. found that the PNPLA3 rs738409 variant was associated with alcoholic liver cirrhosis and elevated aminotransferase levels in Caucasians (14). Mice that received the intestinal microbiota from severe alcoholic hepatitis patients developed more serious liver inflammation, compared to mice harboring intestinal microbiota from no alcoholic hepatitis, which indicated that the gut microbiome contributed to the individual susceptibility of alcoholic liver disease (15). Therefore, we aimed to discover the association between the gut microbiome and individual susceptibility in alcoholic liver damage.

Considering that normal ranges of ALT for men and women were different, and the number of women who drink alcohol is relatively low (16), we only included men in this study. In our study, we found that with similar drinking behaviors (which meant there was no statistical difference in drinking behaviors between the two groups), 23 participants developed elevated aminotransferase, and 55 participants had normal aminotransferase. We hypothesized that the individual susceptibility of alcoholic liver damage is associated with the gut microbiome and firstly used whole-genome sequencing (WGS) to explore the relationship.

## Methods

### Study Population

**Figure 1:**
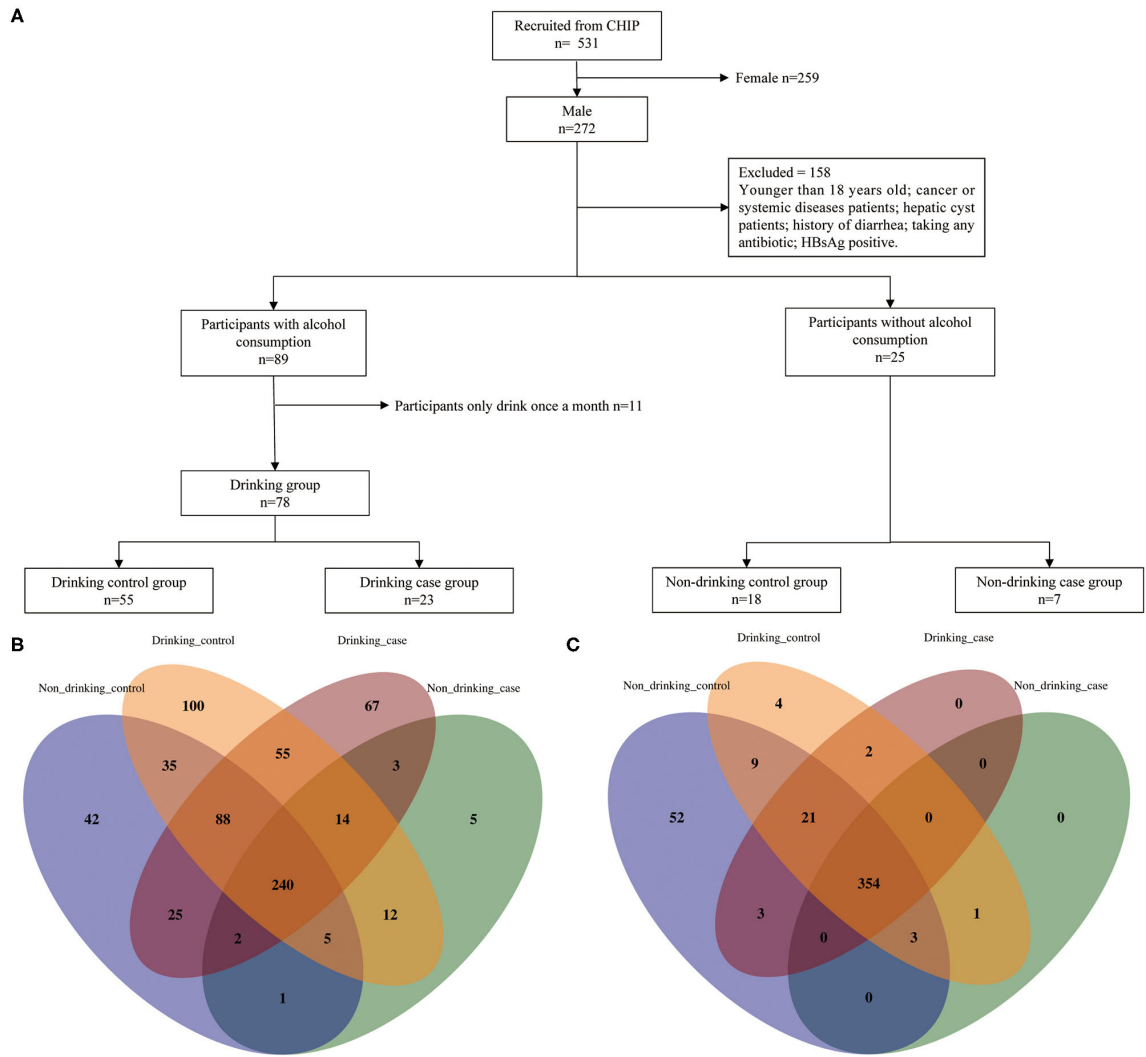
Enrollment flow and distribution of species and metabolic pathways. **(A)** Enrollment flow; **(B)** species distribution; and **(C)** metabolic distribution.

In this cross-sectional study, participants were recruited at the First Affiliated Hospital of Zhengzhou University, Eastern District of the Hospital between January 2018 and April 2019. Totally 531 individuals who had undergone routine physical examinations and had stool specimens collected in the Physician Health Center were included in our study. These were the exclusion criteria: (1) females, (2) participants under 18 years old, (3) cancer or systemic diseases patients, (4) hepatic cyst patients, (5) participants with histories of diarrhea in the previous 3 months, (6) participants who took any antibiotics 3 months prior to the collection of stools, (7) participants who have HBsAg positive illness, and (8) participants who drank alcohol only once a month in the drinking group (Figure 1A).

To clarify how alcohol regulates gut microbiota to cause liver damage, we designed four groups based on two variables: alcohol consumption and liver aminotransferase, namely ALT and AST. The four groups are as follows: (1) non-drinking control group: non-drinkers with normal ALT and AST; (2) non-drinking case group: non-drinkers with elevated ALT or AST; (3) drinking control group: alcohol drinkers with normal ALT and AST; and (4) drinking case group: alcohol drinkers with elevated ALT or AST. The four groups were matched with age and body mass index (BMI).

### Drinking Behaviors Assessment

We collected participants' lifestyle information and personal history including drinking behaviors and smoking status *via* questionnaires. Drinking behaviors were evaluated according to drinking or not, drinking years, drinking frequency, drinking types, and alcohol consumption. If drinking frequency in the questionnaire showed once or twice a month, we calculated it by 1.5 a month. The past week was used as the time period for drinking types and alcohol consumption because individuals could recall their alcohol usage more precisely. Drinking types included liquor, wine, beer, and wine and liquor. Alcohol consumption was assessed based on the alcohol content of various drinking types and consumption of alcoholic beverages. To assess ethanol consumption, we selected the most frequent alcohol by volume for beer, liquor, and wine to calculate ethanol consumption, which are 5, 45, and 12%, respectively. For example, the calculation for beer is: ethanol consumption = beer consumption (ml) × 45% × 0.8 (g/ml).

### Clinical Characteristics Assessment and Stool Sample Collection

Written informed consent was obtained from the participants. Participants were required to fast the night before the physical examination to draw blood tests and collect stool samples. Questionnaires, blood tests, and stool samples were collected on the same day. Blood samples were detected by Roche Cobas 8000 automatic biochemical analyzer (Mannheim, Germany). Both ALT and AST had normal ranges of 0–40 U/L. Stool samples were collected on the day of the physical examination. Indexes controlled attenuation parameter (CAP) and liver stiffness measure (LSM) were assessed by FibroTouch of HISKY Medical Technologies (Wuxi, China). The normal ranges for CAP and LSM are <240 dB/m and <7.3 kPa, respectively. A total of 20 participants performed the FibroTouch examination. The numbers of participants who underwent the examination in non-drinking control, non-drinking case, drinking control, and drinking case were 2, 1, 10, and 7, respectively. We obtained the liver function and FibroTouch information from the management system of the hospital. Stool samples from participants were all freshly collected at the hospital and stored at −80°C within 30 min after subpackaging.

### Genomics DNA Extraction

Following the manufacturer's instructions, DNA from the microbial population was extracted using the MagPure Stool DNA KF Kit B (Magen, Guangzhou, China). DNA was quantified with a Qubit Fluorometer by using the Qubit dsDNA BR Assay Kit (Invitrogen, USA), and the quality was checked by running an aliquot on 1% agarose gel.

### Library Construction

About 1 μg genomic DNA was randomly fragmented by Covaris (Woburn, Massachusetts, USA). The fragmented DNA was selected by magnetic beads to an average size of 200–400 bp. The selected fragments were through end repair, 3′ adenylated, adapters ligation, PCR amplifying, and the products were purified by the magnetic beads. The double-stranded PCR products were heat-denatured and circularized by the splint oligo sequence. The single-strand circle DNA (ssCir DNA) was formatted as the final library and qualified by QC. The qualified libraries were sequenced on BGISEQ 500 platform (BGI, Shenzhen, China).

### Quality Control

Questionnaires and fecal samples were all collected on the same day. Women were excluded from our study due to gender variations in ALT and AST, and gut microbiota. The participants who had undergone physical examinations at the Physician Health Center of the First Affiliated Hospital of Zhengzhou University fasted the night before the examination. We only included drinkers who drank more than once a month to draw a clearer differentiation between the drinking groups and non-drinking groups.

### Statistical Analysis

Statistical analyses were all performed on R version 3.6.1. Continuous variables in demographic characteristics shown as mean (SD) were compared using the Wilcoxon rank-sum test between two groups. Continuous variables in drinking behaviors shown as median (interquartile range, IQR) were compared using the Wilcoxon rank-sum test between two groups. A two-tailed *p*-value < 0.05 was considered statistically significant. The difference of species and pathways of the two groups were calculated by the one-tailed Wilcoxon rank-sum test. Categorical variables, shown as counts and percentages [*n* (%)], were compared using Fisher's exact test, and a two-tailed *p*-value < 0.05 was considered statistically significant. Spearman's rank test was used for the correlation analysis.

### Microbiota Diversity Analysis

The Shannon index, the Simpson index, and the Gini index were used to estimate alpha diversity using the “vegan” package and “ineq” package of R. Beta diversity was assessed by the Pearson distance and Bray–Curtis distance, which were calculated by “vegan” package. We performed principal coordinate analysis (PCoA) to represent the statistically and visually microbial community profile differences using “ade4” package. Alpha diversity indexed and beta diversity distances were performed using the one-tailed Wilcoxon rank-sum test, with the statistically significant *p*-value < 0.05.

Venn diagrams conducted by “VennDiagram” packages were plotted to reveal the common and unique species or pathways in multiple samples and find differential species or pathways, displaying the similarity and overlap among the four groups.

## Results

### Demographic Characteristics of the Cohorts

After a strict inclusion and exclusion process, a totally of 103 participants were enrolled in our study, which included 4 groups: non-drinking control group, non-drinking case group, drinking control group, and drinking case group, containing 18, 7, 55, and 23 participants, respectively (Figure 1A). Participants' age and BMI were all matched among four groups. The clinical characteristics of the four groups are shown in Table 1. The average age of the drinking case group was 38.609 years old. Apparently, ALT and AST were at high levels in the two case groups. Total bilirubin (TBIL), direct bilirubin (DBIL), and indirect bilirubin (IBIL) were all higher in the drinking case group than the drinking control group and non-drinking case group. Globulin tended to be lower in the drinking case group compared to the non-drinking case group. No difference was found in smoking status among the four groups.

**Table 1:** Demographic characteristics of the study participants.

**Feature**	**Non-drinking control**	**Non-drinking case**	**P_1_ value**	**Drinking control**	**Drinking case**	**P_2_ value**	**P_3_ value**
	**(***n*** = 18)**	**(***n*** = 7)**		**(***n*** = 55)**	**(***n*** = 23)**		
**Demographic mean (SD)**							
Age (year)	41.778 (8.257)	41.714 (4.572)	0.855	41.527 (9.309)	38.609 (7.953)	0.273	0.268
BMI (kg/m^2^)	26.772 (3.532)	28.041 (2.713)	0.397	26.856 (3.112)	27.075 (2.773)	0.493	0.327
DP (mmHg)	81.333 (12.551)	81.714 (8.056)	0.738	82.455 (10.136)	80.304 (9.716)	0.316	0.623
SP (mmHg)	128.111 (14.373)	128.429 (6.051)	0.952	131.709 (12.704)	131.043 (15.032)	0.755	0.864
PP (mmHg)	46.778 (8.822)	46.714 (4.309)	0.879	49.255 (8.859)	50.739 (10.037)	0.709	0.476
**Liver function mean (SD)**							
ALT (U/L)	23.222 (7.659)	61.571 (18.347)	<0.001	24.145 (7.077)	59.478 (32.517)	<0.001	0.447
AST (U/L)	20.667 (4.79)	35.857 (14.871)	0.006	21.127 (3.977)	36.174 (19.853)	<0.001	0.768
ALP (U/L)	70.056 (16.232)	65.857 (5.757)	0.363	70.582 (16.156)	72.217 (21.405)	0.852	0.540
GGT (U/L)	25.833 (12.552)	49 (30.111)	0.042	41.345 (33.264)	72.913 (61.854)	0.001	0.404
ALB (g/L)	48.578 (2.537)	50.571 (1.996)	0.034	48.802 (2.287)	49.883 (2.677)	0.143	0.508
GLO (g/L)	26.006 (3.614)	29.714 (3.986)	0.032	26.682 (3.929)	25.87 (4.124)	0.393	0.062
TBIL (μmol/L)	13.829 (5.048)	9.904 (3.185)	0.064	11.792 (3.986)	15.013 (5.897)	0.015	0.029
DBIL (μmol/L)	5.342 (2.135)	4.136 (1.31)	0.244	4.819 (1.277)	5.69 (2.104)	0.070	0.050
IBIL (μmol/L)	8.489 (3.362)	5.757 (2.148)	0.069	6.973 (3.06)	9.326 (4.203)	0.016	0.031
GLU (mmol/L)	5.399 (0.454)	5.659 (1.228)	0.751	5.427 (0.86)	5.364 (0.879)	0.361	0.462
**FibroTouch [*****n*** **(%)]**							
**CAP**							
Normal	0/2 (0.0)	0/1 (0.0)	1.000	2/10 (20.0)	2/2 (28.6)	1.000	1.000
High	2/2 (100.0)	1/1 (100.0)		8/10 (80.0)	5/5 (71.4)		
**LSM**							
Normal	2/2 (100.0)	1/1 (100.0)	1.000	10/10 (100.0)	6/7 (85.7)	0.412	1.000
High	0/2 (0.0)	0/1 (0.0)		0/0 (0.0)	1/7 (14.3)		
**Smoking or not**							
Yes	6/18 (33.3)	1/7 (14.3)	0.626	14/54 (25.9)	7/22 (31.8)	0.587	0.635
No	12/18 (66.7)	6/7 (85.7)		40/54 (74.1)	15/22 (68.2)		

*P_1_, Comparisons between non-drinking control group and non-drinking case group*.

*P_2_, Comparisons between drinking control group and drinking case group*.

*P_3_, Comparisons between non-drinking case group and drinking case group*.

*Continuous variables were compared using the Wilcoxon rank-sum test between two groups. Categorical variables were compared using Fisher's exact test. Statistical analyses were performed using R (Version 3.6.1)*.

*BMI, body mass index; DP, diastolic pressure; SP, systolic pressure; PP, pulse pressure; ALT, alanine aminotransferase; AST, aspartate aminotransferase; ALP, alkaline phosphatase; GGT, gamma-glutamyltransferase; ALB, albumin; GLO, globulin; TBIL, total bilirubin; DBIL, direct bilirubin; IBIL, indirect bilirubin; CAP, controlled attenuation parameter; LSM, liver stiffness measure*.

Table 2 depicts the drinking behaviors of the two drinking groups. We employed some indexes including the drinking years, drinking frequency, drinking type, and ethanol consumption. There was no significant difference in drinking behaviors between the drinking control group and the drinking case group.

**Table 2:** Drinking behaviors.

	**Drinking control**	**Drinking case**	***p*** **-value**
	**(***n*** = 55)**	**(***n*** = 23)**	
Drinking year	15 (7.25–27.5)	15 (10–15)	0.374
Drinking frequency	6 (1.5–6)	1.5 (1.5–6.0)	0.818
**Drinking type**			
Beer	4/33 (12.12)	0/13 (0.00)	0.186
Liquor	27/33 (81.81)	10/13 (76.92)	
Wine	1/33 (3.03)	2/13 (15.38)	
Wine and liquor	1/33 (3.03)	1/13 (7.69)	
Ethanol consumption (g)	54 (36–99)	72 (54–90)	0.438

*Data are median (interquartile range, IQR) or n/N (%). Continuous variables were compared using the Wilcoxon rank-sum test between two groups. Categorical variables were compared using Fisher's exact test*.

### Gut Microbiota Distribution and Diversity of Four Groups

Totally 449 pathways and 694 species (including 215 viruses) were detected in the whole participants. Venn diagrams display the overlaps among the four groups (Figures 1B,C). The four groups shared 240 species and 354 pathways, with 397 species and 377 pathways shared by the drinking control group and the drinking case group. Notably, compared to the other 3 groups, there were 67 species unique to the drinking case group.

**Figure 2:**
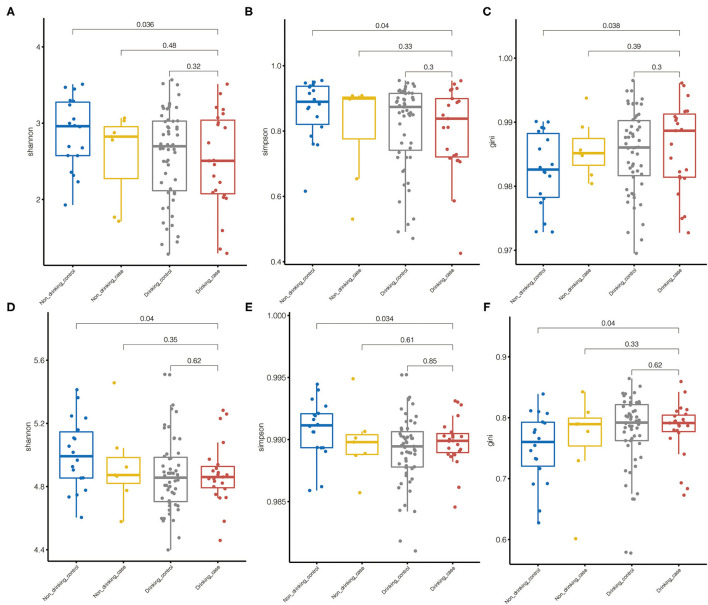
Microbiome diversity. **(A–C)** Species alpha diversity was estimated by the Shannon index, Simpson index, and Gini index, respectively. **(D–F)** Pathway alpha diversity was estimated by the Shannon index, the Simpson index, and the Gini index, respectively.

Species alpha diversity indicated by Shannon index and Simpson index was decreased in drinking case group compared with non-drinking control group. Although there was no statistically significant difference, species Shannon index and Simpson index in the drinking case group tended to be a downward trend in microbiome diversity compared with the non-drinking case group and drinking control group (Figures 2A,B). The Gini index was higher in the drinking case group than that of the other 3 groups, indicating the less equal microbiota distribution for the drinking case group, although only the difference between the drinking case group and non-drinking control group attained statistical significance (Figure 2C). Differences among the non-drinking case group, drinking control group, and drinking case group in alpha diversity were not observed. The alpha diversity of metabolic pathways yielded similar results (Figures 2D–F).

### Altered Overall Gut Microbiota in Drinking Case Group

**Figure 3:**
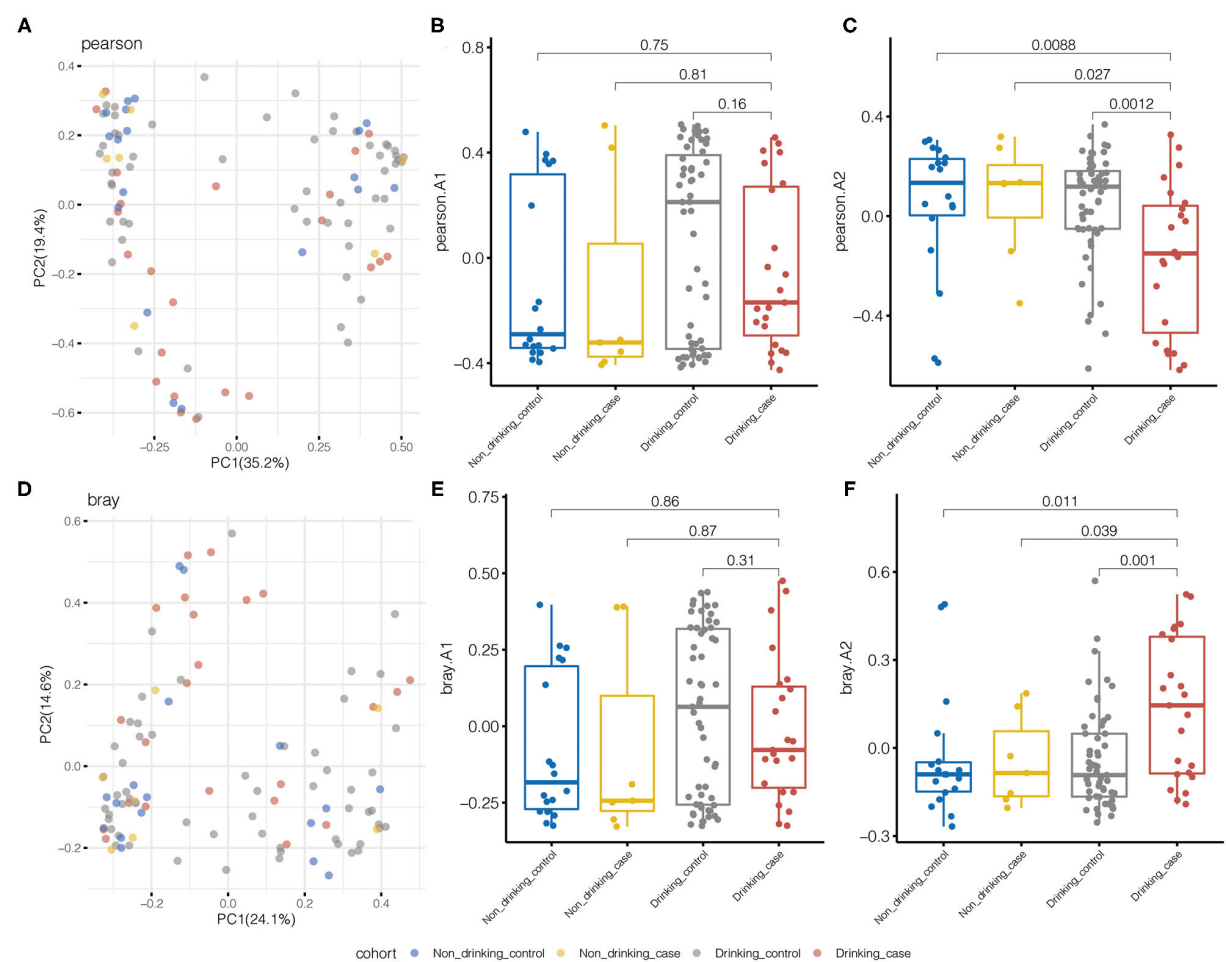
Pearson distance and Bray–Curtis distance illustrated significant differences in the microbial community for species beta diversity. **(A)** Principal coordinate analysis (PCOA) diagram by the Pearson distance. **(B,C)** The first and second principal components based on the Pearson distance. **(D)** PCOA diagram by Bray–Curtis distance. **(E,F)** The first and second principal components are based on the Bray–Curtis distance.

We performed beta diversity calculated by PCOA to display the overall diversity in microbiome composition among four groups. For species diversity, the Pearson distance and Bray–Curtis distance revealed substantial variations in the microbial community between the drinking case group and the other three groups (Figure 3). Samples from the drinking case group (red dots) separated from other groups along the direction of the second axis for the Pearson distance and Bray–Curtis distance, explaining 19.4 and 14.6% of the total variations, respectively.

Metabolic pathway beta diversity was also assessed using the same analysis (Supplementary Figure 1). The drinking case group and the other 3 groups demonstrated significant differences in PCoA based on the Bray–Curtis distance. Drinking case participants could separate from the other three groups along the first axis for the Pearson distance and the second axis for the Bray–Curtis distance. Species and pathway beta diversity confirmed that gut microbial communities were different among the drinking case group and the other three groups.

### Differential Gut Microbiota in the Drinking Case Group

**Figure 4:**
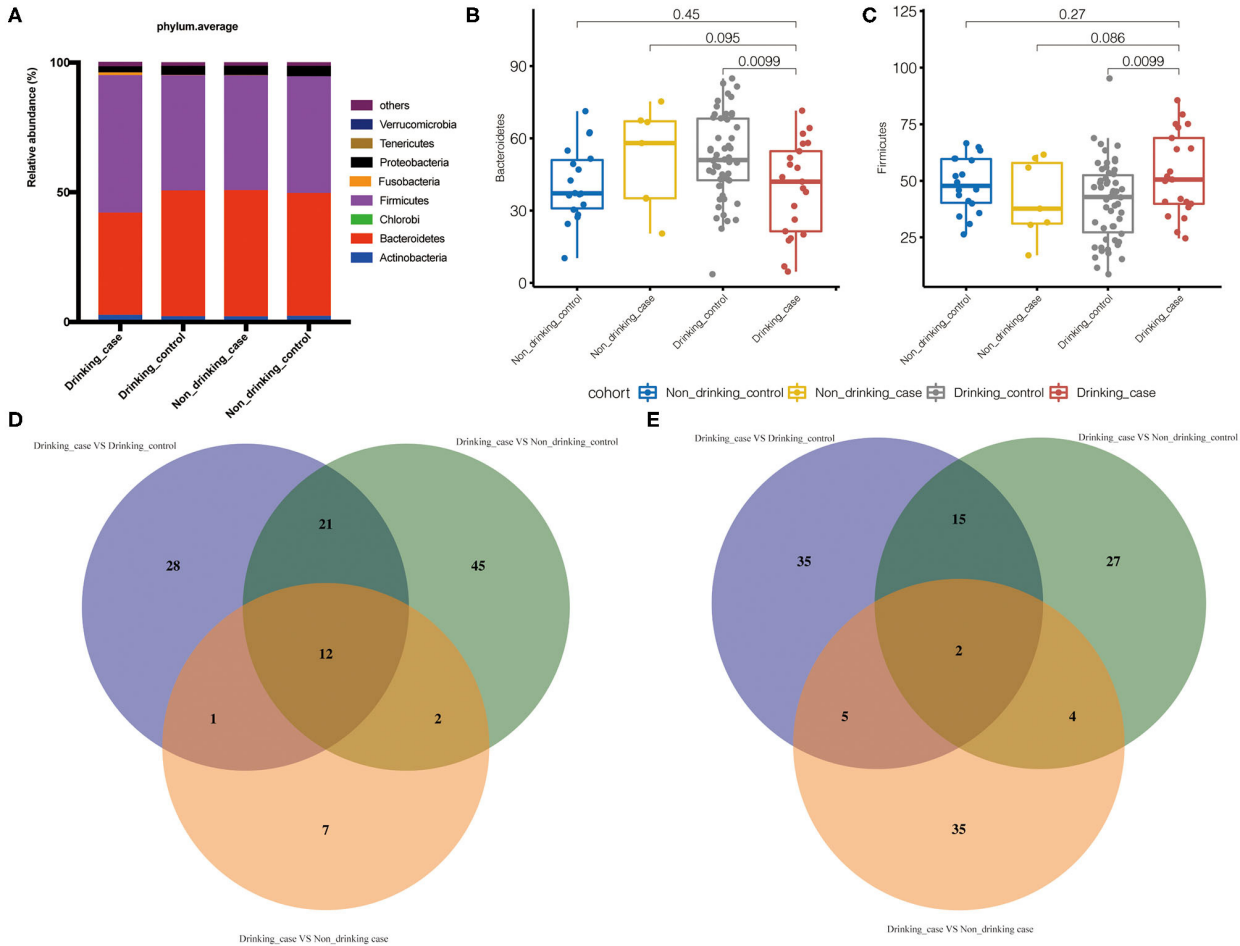
Differential gut microbiota in the drinking case group. **(A)** The average compositions and relative abundance at phylum level of the four groups; **(B,C)** the abundance of phyla *Bacteroidetes* and *Firmicutes* in the four groups; and **(D,E)** the statistically differential overlaps in the distribution of gut microbiome metabolic pathways and species.

To compare the gut microbial communities at phylum and species level among the drinking case group and other three groups, microbial significant differences were analyzed by the one-tailed Wilcoxon rank-sum test. The average compositions and relative abundance at the phylum level were displayed (Figure 4A). Apparently, phylum *Bacteroidetes* was reduced in the drinking case group vs. the drinking control group, whereas phylum *Firmicutes* was increased (Figures 4B,C).

To demonstrate the difference at the species level, we further used Venn diagrams to show the statistically differential overlaps in the distribution of gut microbial species and metabolic pathways (Figures 4D,E). Overall, there were 2 differential species and 12 pathways related to the drinking case group. Furthermore, there was no statistical difference among the non-drinking control group, non-drinking case group, and drinking control group in the 2 species and 12 pathways (Supplementary Table 1).

**Figure 5:**
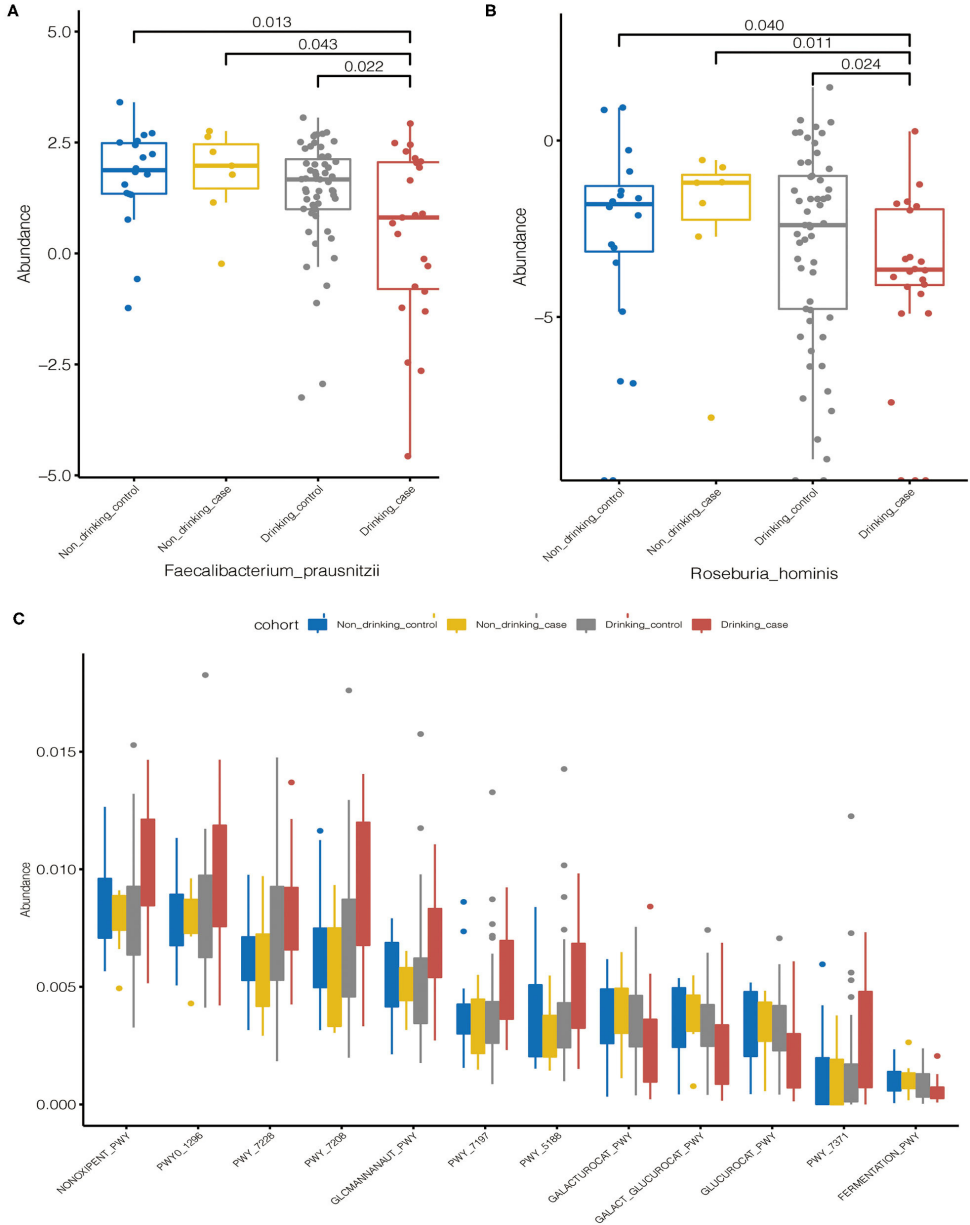
Statistically differential species and metabolic pathways among the four groups. **(A,B)** The distribution of the statistically differential species (*Faecalibacterium prausnitzii* and *Roseburia hominis*); **(C)** The distribution of the statistically differential metabolic pathways.

The distribution of the statistically differential species and metabolic pathways among the four groups was demonstrated in the Boxplot diagrams (Figure 5). Compared to the other three groups, the drinking case group had a lower abundance of differential species, *Faecalibacterium prausnitzii*, and *Roseburia hominis*. Eight of the 12 differential metabolic pathways were enriched in the drinking case group, and the other 4 pathways were decreased in the drinking case group. For the 8 pathways enriched in the drinking case group, three are associated with nucleoside and nucleotide biosynthesis (PWY-7197, PWY-7208, and PWY_7228), one is associated with amine and polyamine degradation (GLCMANNANAUT-PWY), one is associated with pentose phosphate pathways (NONOXIPENT-PWY), one is associated with carrier biosynthesis (PWY_7371), one is associated with tetrapyrrole biosynthesis (PWY_5188), and one is associated with nucleoside and nucleotide degradation (PWY0-1296). For the four pathways decreased in the drinking case group, one is associated with galacturonate and glucuronate catabolism (GALACT-GLUCUROCAT-PWY), one is associated with β-D-glucuronide degradation (GLUCUROCAT-PWY), one is associated with D-galacturonate degradation (GALACTUROCAT-PWY), and one is associated with mixed acid fermentation (FERMENTATION-PWY).

### Correlations Between the Gut Microbiome and Liver Damage

**Figure 6:**
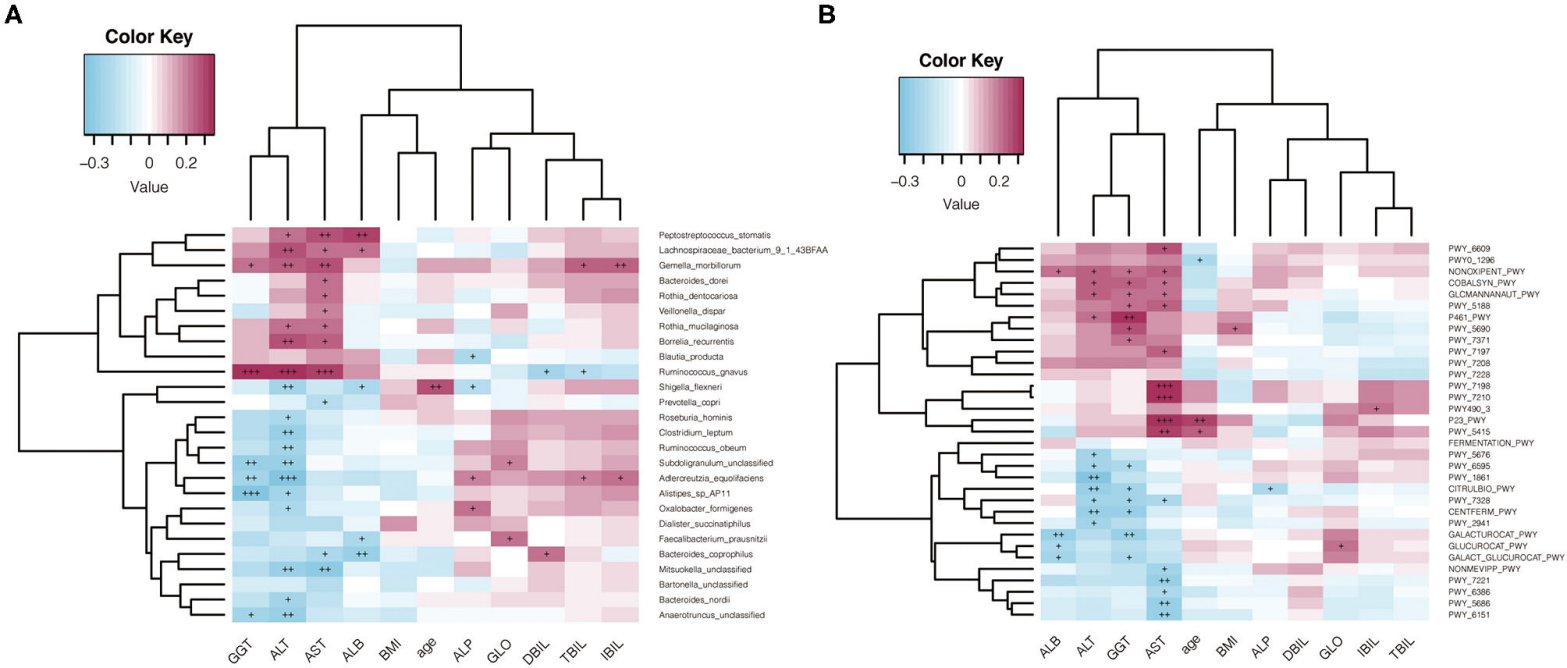
Spearman correlation analysis. **(A)** Spearman analysis of species abundance and clinical indexes; **(B)** Spearman analysis of metabolic pathways and clinical indexes.

To identify the correlations between clinical variables and microbiota, associations between the gut microbiome and continuous clinical variables were calculated by the Spearman correlation coefficient in all participants. Species heatmap revealed the correlation between species and clinical variables (Figure 6A). *R. hominis* showed a negative correlation with ALT and significantly decreased in the drinking case group. Among all the species in the heatmap, *Ruminococcus gnavus* showed the most positive correlation with ALT and AST.

For pathways, we noticed that NONOXIPENT-PWY, GLCMANNANAUT-PWY, PWY-5188, and PWY-7197 were all positively correlated with ALT or AST, and they were increased in the drinking case group (Figure 6B).

## Discussion

We firstly performed WGS in the comprehensive groups to explore the association between the gut microbiome and individual susceptibility in alcohol-related liver transaminase elevation. In our study, although the two drinking groups showed no differences in drinking behaviors, one group had raised transaminases and the other group had normal transaminases—indicating individual variability reflecting the liver injury caused by alcohol consumption. In our study, we observed fecal microbiome community in the drinking case group differed from the other groups and described the significantly different species and metabolic pathways.

Liver function indexes ALT and AST are the easiest markers to assess hepatocellular damage and usually are the screen tests for liver disease (5). Compared to ALT, AST is more widely distributed in many tissues—including liver, skeletal muscle, heart, etc. (17). Considering the weak specificity of AST distribution, we ruled out participants with cancers, any systemic diseases, and any patients with liver disease. ALT and AST also can be influenced by genetic contribution (18), gender differences (19), exercise (20), late sleep, and other environmental factors. Men have higher levels of ALT and AST than women, according to the large cohort studies (16, 21). Considering the possible analytical errors, we only included male participants in this study and also established the non-drinking case group (participants who had no alcohol consumption with elevated ALT and AST).

Microbiome alpha diversity of the drinking case group based on the Shannon index and the Simpson index was lower compared to HC participants. Alcohol use disorder patients were found to have a decreased alpha microbiome diversity in the former study (22). The reduction microbial diversity was one of the major types of gut disease-associated dysbiosis (23) and was documented in many diseases, such as IBD (24), autoimmune hepatitis (25), and type 1 diabetes (26). The prior research proved that individuals with decreased microbial richness have more tendency to develop low-grade inflammation (27, 28). The alcohol consumers who have elevated ALT and AST may develop dysbiosis in gut microbiome composition and more possibility to develop inflammation.

In our study, we found elevated phylum *Firmicutes* and decreased phylum *Bacteroidetes* in the drinking case group compared to the drinking control group. Phyla *Firmicutes* and *Bacteroidetes* are dominant and account for the majority of human gut microbiota (29). Former studies reported lower *Bacteroidetes* in individuals with alcoholism and cirrhosis, which was consistent with our findings (8, 30). Ethanol treatment in mice induced the decrease of *Firmicutes* and *Bacteroidetes*, especially *Bacteroidetes* (31). *Bacteroidetes* was found to be significantly reduced following the ethanol feeding to mice in this 8-week investigation. The different findings of phylum *Firmicutes* may be the result of the difference of observed objects and events.

The intersections of bacteria with statistical differences were obtained using the Venn diagram, and finally, two species were obtained, which were *F. prausnitzii* and *R. hominis*, and both were significantly reduced in the drinking case group. Increased gut permeability permits the translocation of macromolecules including endotoxins, contributing to the alcoholic liver damage (11). *F. prausnitzii* and *R. hominis* are both important butyrate producers and contribute to gut integrity (32). *F. prausnitzii* is one of the most common bacteria in human gut flora (33). Microbial anti-inflammatory molecule (MAM) was discovered to be the production of *F. prausnitzii* and could inhibit the NF-κB pathway in the intestinal epithelial cells, therefore, preventing mice from colitis (34, 35). Activation of NF-κB pathway participates in the breakdown of the intestinal barrier caused by alcohol (36). *R. hominis* significantly decreased in the drinking case group and also showed a negative correlation with ALT. *R. hominis* treatment could relieve colitis and reduce inflammatory markers including interleukin (IL)1-β, IL6, and tumor necrosis factor (TNF)-α (37). TNF-α is an important inflammatory cytokine and can promote alcoholic liver damage, mostly produced by liver Kupffer cells (38, 39). *Ruminococcus gnavus* showed the most positive correlation, both with ALT and AST. *Ruminococcus gnavus* was reported as a potential pathogen in infectious disease and exhibited an increased abundance in inflammatory bowel disease (40, 41). In 2019, a study reported that *Ruminococcus gnavus* was the producer of inflammatory polysaccharides, a promotor in TNF-α secreted by dendritic cells (42). Therefore, *R. hominis* and *Ruminococcus gnavus* showed opposite effects on the TNF-α production. Alcohol enhances the sensitivity of Kupffer cells to product TNF-α (37, 43), which can be decreased by *R. hominis* and increased by *Ruminococcus gnavus*.

We discovered 12 metabolic pathways that were statistically different among the drinking case group and the other three groups. Among the eight pathways, which increased in the drinking case group, 3 pathways were associated with nucleoside and nucleotide biosynthesis and 1 pathway was associated with nucleoside and nucleotide degradation. The activation of the processes might associate with the dysbiosis of gut microbiome and the possible bacterial overgrowth in patients with alcoholic liver injury (44, 45). The other alterations of metabolic pathways may be generated by the dysbiosis of the gut microbiome.

These are the advantages that only the participants without underlying diseases were included, and we designed four groups based on the alcohol consumption and liver aminotransferase. However, due to the strict exclusion criteria, the number of subjects was limited, and we only included male participants. Some participants forgot their drinking behavior resulting in missed information. In our study, the most important was that we discovered three altered species, which were related to alcoholic liver injury. The finding should be further verified through large-scale studies and animal experiments.

## Conclusion

Alcoholic liver damage is usually noted early in the clinic with elevated aminotransferase levels. Our findings suggested that the gut microbiome contributes to the susceptibility of individuals to develop liver injury after alcohol consumption. Species *F. prausnitzii* and *R. hominis* exhibited a protective effect on the liver, and *Ruminococcus gnavus* showed a liver-damage effect. Further verification is needed in future studies. Since the three meaningful species were discovered in this study, we believe that after further verification, the probiotic administration or dietary patterns can be used to regulate intestinal flora, and thus protect the liver of alcohol drinkers.

## Data Availability Statement

The datasets presented in this study can be found in online repositories. The names of the repository/repositories and accession number (s) can be found below: https://db.cngb.org/search/project/CNP0002336/.

## Ethics Statement

The studies involving human participants were reviewed and approved by Ethics Review Committee of Scientific Research Projects in The First Affiliated Hospital of Zhengzhou University. The ethics approval number is 2018-KY-56. The patients/participants provided their written informed consent to participate in this study.

## Author Contributions

AL, SD, and JL contributed to the conception and design of the study. MJ performed the analysis and wrote the manuscript. MJ, SY, and AL performed the data analysis. MJ and JL analyzed the participants' data. QS, YLiu, and YLi helped collect the data and edit the manuscript. All authors read and approved the final manuscript.

## Funding

This study was equally funded and supported by the Henan Province Key Science and Technology Program 172102310049, the Henan Province Medical Science and Technique Project 2018020001 Grant, and the Henan Province Postdoctoral Research Grant 001801005.

## Conflict of Interest

The authors declare that the research was conducted in the absence of any commercial or financial relationships that could be construed as a potential conflict of interest.

## Publisher's Note

All claims expressed in this article are solely those of the authors and do not necessarily represent those of their affiliated organizations, or those of the publisher, the editors and the reviewers. Any product that may be evaluated in this article, or claim that may be made by its manufacturer, is not guaranteed or endorsed by the publisher.

## Supplementary Material

Supplementary Figure 1 Pearson distance and Bray–Curtis distance illustrated significant differences in the microbial community for pathway beta diversity. **(A)** Principal coordinate analysis (PCOA) diagram by the Pearson distance. **(B,C)** The first and second principal components are based on the Pearson distance. **(D)** PCOA diagram by the Bray–Curtis distance. **(E,F)** The first and second principal components are based on the Bray–Curtis distance.

Supplementary Table 1 Statistically differential species and metabolic pathways related to the drinking case group.
