# Supplementary figures and images for: Alcohol-Related Elevation of Liver Transaminase Is Associated With Gut Microbiota in Male
Source: Front Med (Lausanne). 2022 Feb 22;9:823898. doi: 10.3389/fmed.2022.823898 (PMC8904186; doi:10.3389/fmed.2022.823898)

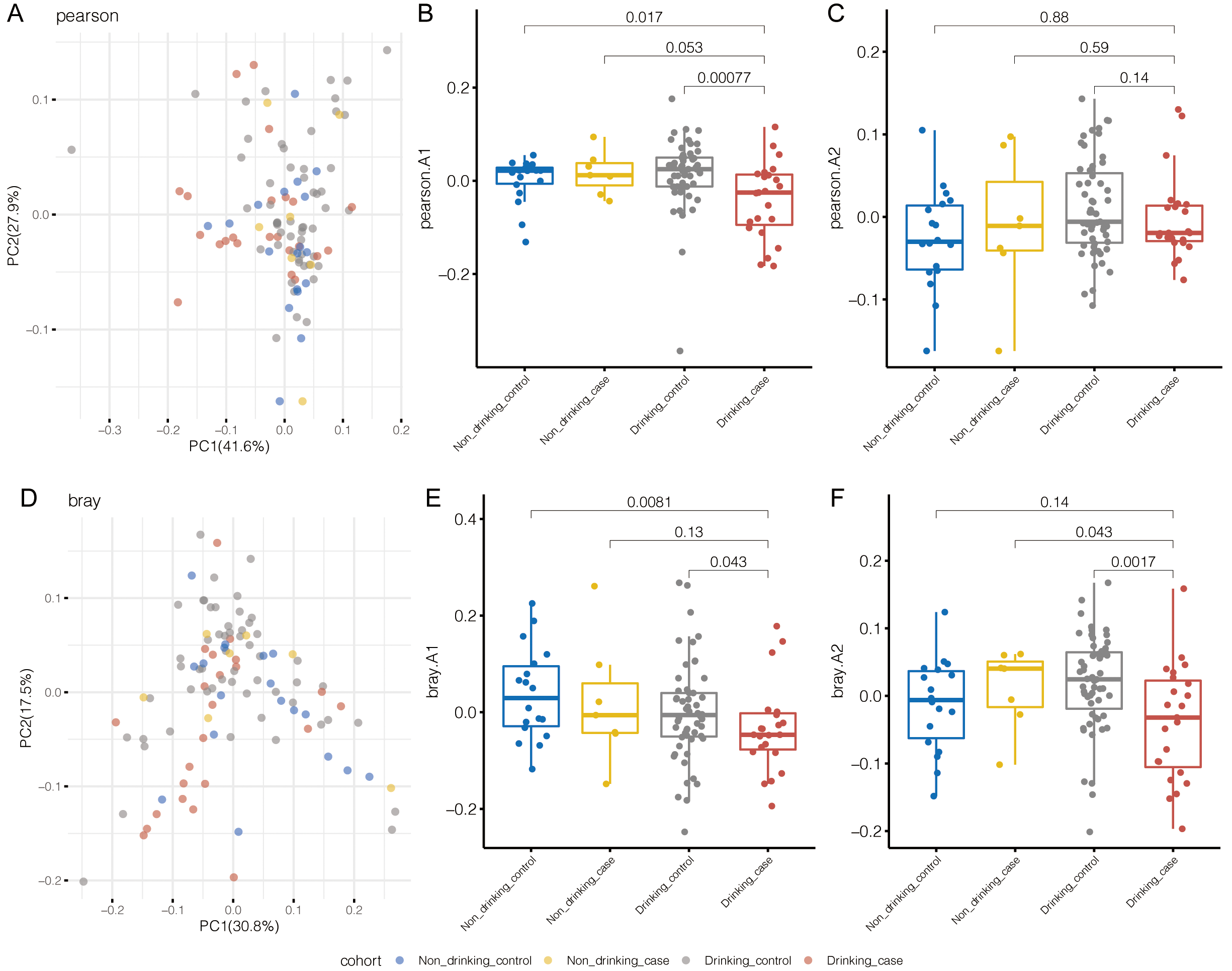

Supplement: Supplementary Figure 1 — Pearson distance and Bray–Curtis distance illustrated significant differences in the microbial community for pathway beta diversity. (A) Principal coordinate analysis (PCOA) diagram by the Pearson distance. (B,C) The first and second principal components are based on the Pearson distance. (D) PCOA diagram by the Bray–Curtis distance. (E,F) The first and second principal components are based on the Bray–Curtis distance. [file Image_1.TIF]
